# Training in women mental health: Challenges and future perspectives

**DOI:** 10.1192/j.eurpsy.2021.191

**Published:** 2021-08-13

**Authors:** A. Wieck

**Affiliations:** Greater Manchester Mental Health Foundation Trust, University of Manchester, Manchester, United Kingdom

**Keywords:** training, Womens mental health

## Abstract

Mental disorders linked with the menstrual cycle, childbirth and the menopause are unique to women and have their specific requirements for identification, assessment, treatment and service provision as well as appropriately trained staff. Mental disorders that are linked to domestic violence, sexual abuse or victimisation and affect more women than men, also have their own specific requirements. This is increasingly being recognized by policy makers and health care planners. In the case of perinatal mental health some countries have set up specialised inpatient and community services. In the UK this was followed by the development of National competencies for different professional groups and National training programmes. This project and the current clinical training in perinatal mental health for psychiatric trainees will be described. Nationally recognized qualifications are currently being developed. Developments in other areas of women’s mental health appear less advanced. The presentation will discuss current guidelines and opportunities for training that are being offered in the UK and what we can learn from perinatal mental health for further development in these areas.

**Disclosure:**

No significant relationships.

